# Comparison of Ni-Based Self-Fluxing Remelted Coatings for Wear and Corrosion Applications

**DOI:** 10.3390/ma14123293

**Published:** 2021-06-14

**Authors:** Norbert Kazamer, Roxana Muntean, Petru Cristian Vălean, Dragoș Toader Pascal, Gabriela Mărginean, Viorel-Aurel Șerban

**Affiliations:** 1 Westphalian Energy Institute, Westphalian University of Applied Sciences Gelsenkirchen Bocholt Recklinghausen, Neidenburgerstr. 43, 45897 Gelsenkirchen, Germany; dragos.pascal88@gmail.com; 2 Department of Materials and Manufacturing Engineering, University Politehnica of Timișoara, Piața Victoriei 2, 300006 Timișoara, Romania; roxana.muntean@upt.ro (R.M.); petru_valean@yahoo.com (P.C.V.); viorel.serban@upt.ro (V.-A.Ș.); 3 Department of Materials Science and Testing, Westphalian University of Applied Sciences Gelsenkirchen Bocholt Recklinghausen, Neidenburgerstr. 43, 45897 Gelsenkirchen, Germany; gabriela.marginean@w-hs.de

**Keywords:** flame-spraying, NiCrBSi, vacuum furnace, remelting, microstructure, phase composition, wear resistance, corrosion behavior

## Abstract

The present study investigates the possibility to apply a vacuum furnace thermal post-treatment as an alternative solution for flame sprayed NiCrBSi wear and corrosion-resistant coatings, deposited on a low alloyed structural steel. The controlled atmosphere offers advantages regarding the fusion of the coating, porosity reduction, and degassing. An improvement of the applied heating-cooling cycle was performed through the variation of time and temperature. The best performing samples were selected by comparing their porosity and roughness values. The chosen samples were subsequently characterized regarding their microstructure, microhardness, sliding wear, and corrosion behavior. The experimental work confirms that the use of a vacuum remelting post-process reduces the porosity below 1% and leads to the formation of a larger quantity of hard boron-containing phases, promoting a significant decrease of the wear rate, while maintaining a good corrosion behavior.

## 1. Introduction

NiCrBSi coatings in various chemical compositions are predominantly used in paper, petrol, hot working punches, heat exchangers, or glass mold industries [1,2,3,4]. Containing borides and carbides, distributed within the microstructure, the Ni-based alloys show excellent resistance during wear tests [3]. The superior corrosion behavior is attributed to the presence of chromium, as an alloying element in the coating. These compositions are often used as a substitute for hard chromium coatings, well known for environmental issues [5]. Nevertheless, despite the technological advances, flame sprayed Ni-based alloy coatings generally manifest moderate adhesion to the substrate, high porosity, unmelted particles, and unwanted oxides [6]. Subsequently, a fusing process is imperatively necessary to eliminate the undesired microstructural features resulted from spraying technology and consequently to improve the properties and characteristics of such coatings [6]. The presence of boron and silicon lowers the melting temperature, making this alloy a suitable candidate for applying remelting processes [2].

The best known technologies for the post-treatment are flame, furnace, laser remelting, and induction [2,6,7,8,9]. The most commonly applied technology is flame remelting because of the low cost and ease of handle. However, the relative low control of the process, risk of heat-affected zones creation, and uncontrolled atmosphere make this process, in certain situations, undesirable to use [10]. Lately, vacuum furnaces have been considered for heat treatments, as they can offer service stability due to the nature of the processing environment, possibility of handling parts with complex geometries, and relatively low costs, with a short recoupment period [11]. A controlled atmosphere helps to significantly improve the quality of the surface coating by realizing gas extraction, eliminating pores, and offering reproducible results [12,13].

The aim of this research work is to investigate the influence of vacuum furnace post-treatment of Ni-based thermally sprayed coatings. The novelty of the research is represented by the remelting process of the proposed alloy, in a vacuum furnace, correlated with a comparison with the classical method of flame remelting, in respect to its resistance during different tests. The microstructure, phase composition, microhardness, porosity, adhesion of both flame remelted (FR), and vacuum remelted (VR) coatings were investigated, and the wear and corrosion behavior were evaluated according to the specific standards.

## 2. Materials and Methods

### 2.1. Feedstock Powder and Methods for Characterization

**Figure 1:**
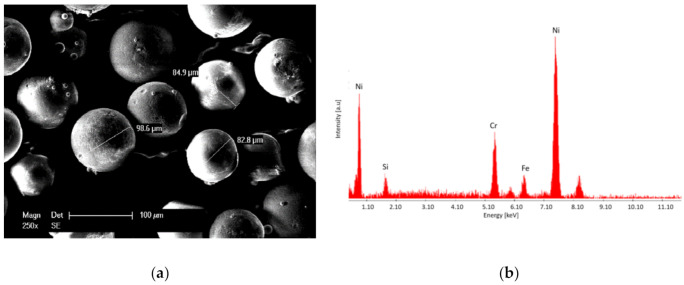
SE micrograph (**a**) and EDX spectrum (**b**) of the NiCrBSi powder.

The SEM (scanning electron microscopy, Philips XL 30, Eindhoven, The Netherlands) micrograph shown in Figure 1a presents the spheroidized shape and the size of approximately 90 µm of the NiCrBSi powder, while Figure 1b demonstrates, based on the EDX analysis, the high Ni content of the proposed alloy. The chemical composition of the powder can be seen in Table 1. The EDX spectrum (EDAX AMETEK, Mahwah, NJ, USA) highlights the carbide forming element, Cr, which contributes to an increase in the wear resistance, and Si, which promotes the deoxidation and wettability of the coating during the heat treatment. The spheroidized morphology of the powder is a favorable aspect during the deposition process, assuring the flowability of the particles.

### 2.2. Coating Deposition and Remelting

The flame spraying of the NiCrBSi coatings was performed with the parameters displayed in Table 2 by the partner company Karl Schumacher GmbH, Germany. The work piece was subjected to degreasing and grit-blasting, to obtain a good tensile adhesive strength and prevent strong substrate oxidation [14].

**Figure 2:**
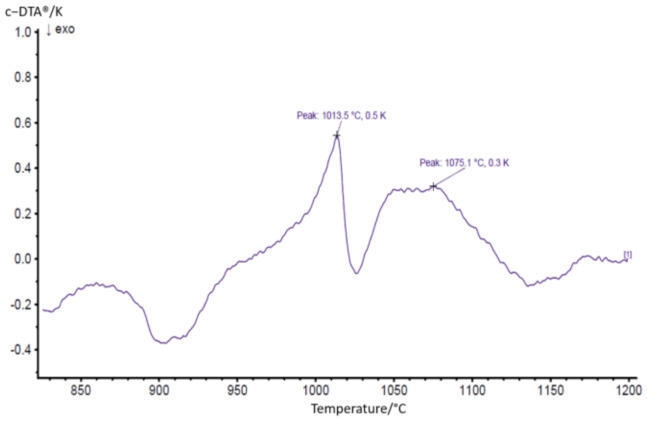
DTA curve highlighting the solidus and liquidus temperature of the NiCrBSi powder.

Preceding the remelting process, a careful thermal analysis of the powder was performed with the aid of a Netzsch STA 449 F1, instrument, NETZSCH, Selb, Germany. On the DTA (differential thermal analysis) curve, represented in Figure 2, a melting interval between 1013 °C and 1075 °C has been identified. In order to achieve densification of the coating and to prevent the liquid-phase flow during the thermal post-treatment, it is important to choose a temperature with 10°C –20 °C under the liquidus temperature, as determined from the thermal analysis [15]. 

**Figure 3:**
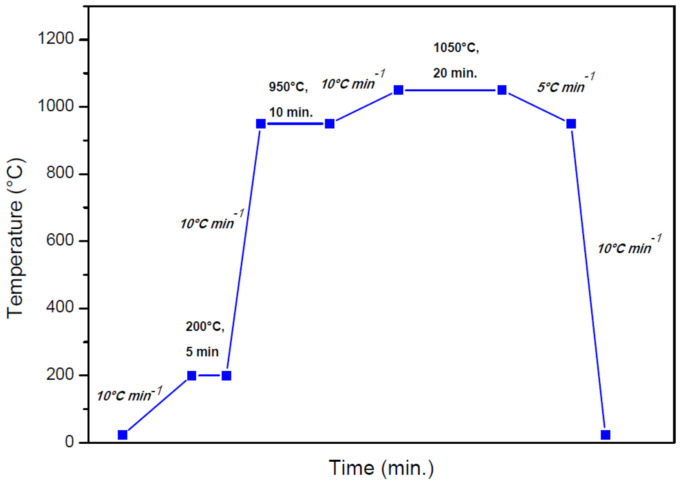
Vacuum furnace heating/cooling cycle.

The remelting process of the coatings was performed with the help of a HITERM 80-200, HITEC Materials, Karlsruhe, Germany, vacuum furnace. Figure 3 represents the cyclogram of the applied thermal treatment, where the ramps and holding times were set corroborating the DTA data with empirically adjusted parameters. The program has three-interval heating ramps, the first being performed with 10 °C min^−1^ up to 200 °C, where water and other possible compounds can be eliminated from the sample’s surface. The second heating ramp is performed with 10 °C min^−1^, until 950 °C. At this temperature, the sample is under the melting range of the matrix, but high enough to soak the material. A slow heating step at 5 °C min^−1^ has been applied, followed by a holding of 20 min. at 1050 °C, as resulted from the DTA data. Furthermore, a slow cooling until 950 °C was performed to avoid the occurrence of internal stresses. The last cooling ramp was set at 10 °C min^−1^ until the ambient temperature of 23 °C was reached.

The reference specimens for the present study consisted of identical thermal as-sprayed samples, flame remelted with the Metatherm, gun using the same gas stoichiometry of C_2_H_2_:O_2_ 1:2. Due to the tendency of energy dissipation, the torch was first moved near the coated samples and the material was preheated at 500 °C ± 2 °C. The flame remelting was performed at a temperature in the melting range of the material, as determined with the DTA analysis, by moving the gun as a translation over the samples for approximately 10 min. The temperature was monitored using an infra-red thermometer.

### 2.3. Sample Roughness Measurement and Porosity Estimation

The sample roughness measurements were carried out in three areas of each specimen with the help of a dedicated software, using a CLSM Keyence VK-X microscope, Japan. For this analysis, the two most important parameters that were considered for the characterization of the surface roughness were *Ra* and *Rz*, as defined by Whitehouse [16]. The porosity of the coatings was estimated on three different regions from the optical micrographs, using the open source image analysis software, ImageJ (version 1.53j).

### 2.4. Microstructure, Hardness, Adhesion, Wear, and Corrosion Testing

Several tests were undertaken to investigate the quality of the coating. The morphology, microstructure, and chemical composition were studied using a XL 30 SEM, Philips, Eindhoven, The Netherlands, combined with its EDX detector. The micrographs were acquired at a 10 mm working distance and a cathode voltage of 25 kV. The phase composition was analyzed using the X’Pert X-ray diffractometer, Philips, Eindhoven, The Netherlands. The measurements were performed in the reflection mode, with CuKα as the radiation source, at 40 kV and 40 mA, scan rate of 0.01° 2θ min^−1^, and an angular resolution of 0.005° 2θ. The hardness evaluation of the coatings was performed using a Zwick/Roell ZHV µ-S Vickers microhardness tester, Ulm, Germany, with a total load of 2.94 N (HV0.3), in five different indentations on each specimen. As similarly tested by Tunis et al. [16], the adhesion at the interface was examined through three sets of Brinell macroindentations, under three different loads, with a maximum load of 1838 N (HBW 2.5/187.5). Pin-on-disk tests were realized on a CSM Instruments Tribometer, Needham Heights, MA, USA, and the applied parameters are shown in Table 3. The tests were undertaken to measure the coefficient of friction, and subsequently, the wear rate coefficient of the sample was calculated using the Archard equation:

(1) $$k=\frac{W_{v}}{F_{N}\cdot s}$$

where the wear rate coefficient *k* (mm^3^ N^−1^ m^−1^) is the wear volume *W_V_* (mm^3^) divided by normal load *F_N_* (N) multiplied with the sliding distance *s* (m) [17]. The counterbody (6 mm WC-Co ball) worn volume *W_V_* was considered as well. The corrosion tests were carried out in a three-electrode cell, using 3.5% NaCl standardized solution, at room temperature. The applied potential varied between −1000 and +500 mV versus Saturated Calomel Electrode (SCE), with a scan rate of 0.16 mV s^−1^. The tests were realized using a VoltaLab PGP201 Potentiostat/Galvanostat, Radiometer Analytical SAS, Lyon, France. For reproducibility, each investigation was performed three times.

## 3. Results

### 3.1. Sample Roughness and Porosity Calculation

**Figure 4:**
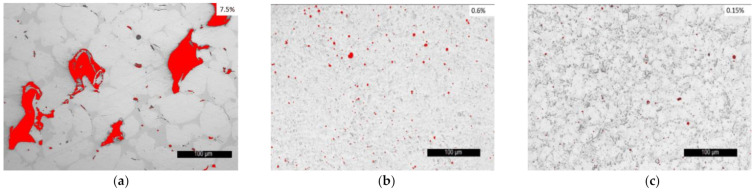
Porosity values of the, as-sprayed (**a**), FR (**b**), and VR (**c**) samples.

It is important to consider the surface roughness and porosity of the functional coatings. At high porosity values, the penetration of water molecules, oxygen, or other unwanted compounds into isolated regions of inhomogeneities is more likely than at smaller values, resulting in unsatisfactory corrosion behavior of the material [18]. During the optimization process in the vacuum furnace, it was noticed that the samples kept for a longer period of time at 1050 °C, present a shinier and smoother surface, being also an important indicator that the surface is completely sealed. The roughness measurements showed that both *Ra* and *Rz* average values, which can be found in Table 4, for the VR samples are approximately 30% lower in comparison with those of the FR coatings. Regarding the porosity, also presented in Table 4, it is interesting to notice that its value decreased from 7.55% in the case of the flame-sprayed coatings to 0.6 % for the flame remelting process and it reached a value of 0.15% for the VR samples. The porosity of the as-sprayed coatings is shown in Figure 4a, and for the post-treated samples is highlighted in Figure 4b,c. The wetting properties and the surface tension of the phases can be controlled through the content of silicon and boron [19], directly affecting the porosity and acting as fluxing agents. The decrease of porosity in the case of the VR sample can be attributed to the capacity of the furnace to precisely control and maintain the temperature of the sample at a few degrees lower than the liquidus point of the alloy, which leads to a proper wetting of the substrate surface, a good degassing, and closure of voids.

**Figure 5:**
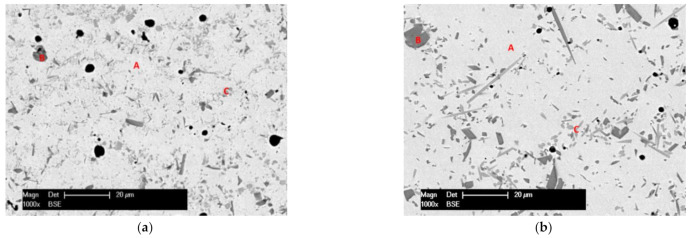
BSE micrograph of a detail of the FR (**a**) and VR (**b**) coatings.

**Figure 6:**
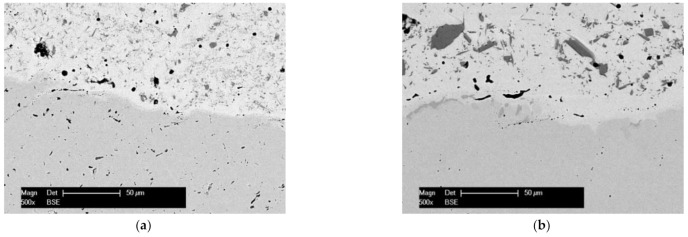
BSE micrograph of the interface at the FR (**a**) and VR (**b**) samples.

**Figure 7:**
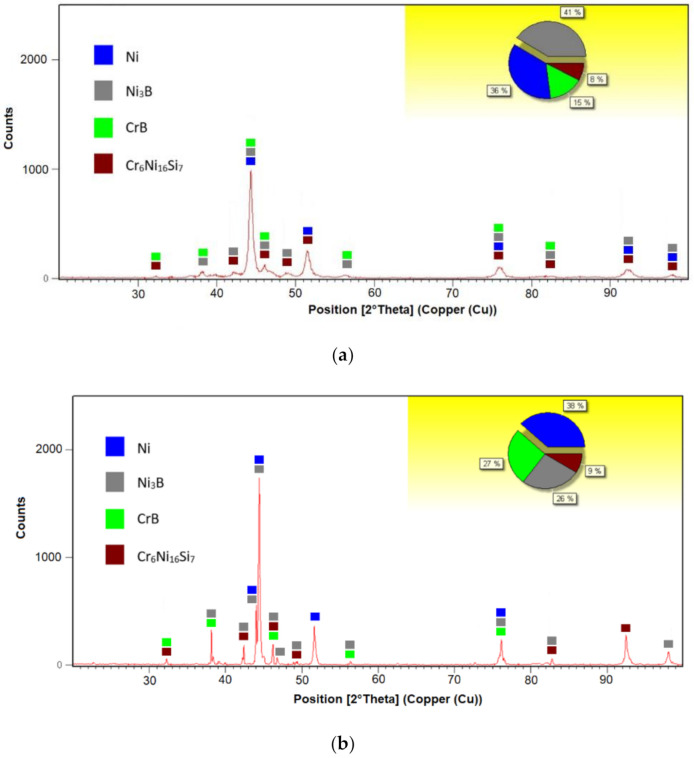
XRD spectra of the FR (**a**) and VR (**b**) samples.

The microstructure was carefully analyzed for both remelted specimens. The EDX spot analysis was focused on identifying only the chemical elements Ni, Cr, Si, and Fe, whereas B and C are too light, and their weight percentage is too low to be detected with this method. It is important to consider that the cooling rate of the particles during the thermal spraying processes is higher than 10^6^ K s^−1^, causing metastable microstructures [20]. The phase composition determined by means of XRD analysis consists, in both cases, of γ-Ni/Ni_3_B solid solution and further phases like CrB and Cr_6_Ni_16_Si_7_. Similar γ-Ni/Ni_3_B phases were already previously reported in the literature [21,22,23]. Corroborating the XRD with the EDX spot analysis, the matrix marked with A in Figure 5a,b consists of the γ-Ni/Ni_3_B phase, the darker region marked with B shows the primary crystals of CrB, while the lighter region of C represents the Cr_6_Ni_16_Si_7_. It has been suggested that CrB forms during the primary solidification [6] if the content of B in the powder exceeds 0.8% [24]. The interface region of the coatings, Figure 6a,b, shows no evidence of delamination or cracks. A higher concentration of the CrB can be seen in the region of the interface. That is caused by the fact that the phase has a higher density than that of the molten phase, which brings the hard phase through gravity towards the coating-substrate area. In the region of the interface, a dilution of Ni appears towards the substrate. Looking closer at the XRD spectra, presented in Figure 7a,b, it can be seen that the quantity of Ni and Cr_6_Ni_16_Si_7_ phases is similar, while the amount of CrB increases in the VR sample. The impact of the phase quantity directly affected the coating behavior during the sliding wear tests.

### 3.2. Hardness and Adhesion

**Figure 8:**
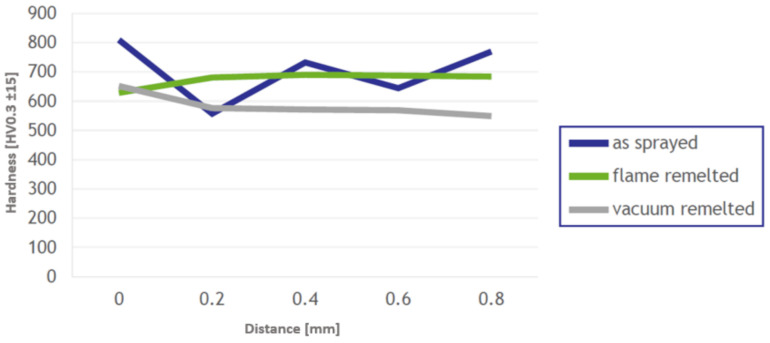
Graph highlighting the variation of the HV0.3 microindentations along the coating.

The microhardness of the coatings was evaluated by realizing one set of imprints consisting of five indentations along the coating on each specimen and the distribution of the values is presented in Figure 8. Although the as-sprayed coating exhibits the highest value of 809 HV0.3, it also shows a variation of over 30% between the maximum and the minimum point of the coating hardness, the difference being accounted to the pores and material inhomogeneities. Concerning the remelted samples, the FR specimen exhibited an average value of 674 HV0.3, while the VR one, an average value of 583 HV0.3. The indentation sets manifested constant values, the difference between the extreme points not exceeding 10%, aspect denoting a homogeneous coating, with a relatively uniform phase distribution. The difference in the microhardness values may lie in the cooling rates of the two remelting approaches, where the uncontrolled atmosphere of the flame-remelting technology favors a much faster cooling of the samples, while the vacuum furnace installation confers a controlled cooling rate, the grain growth is continuous, hence smaller hardness values are obtained.

**Figure 9:**
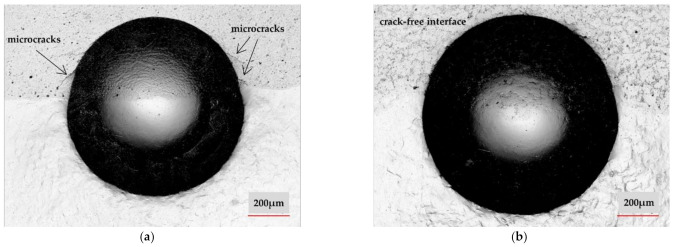
HBW 2.5 / 187.5 on: FR (**a**) and VR (**b**) samples at the coating/substrate interface.

Regarding the adhesion of the coating to the substrate, submitting the interface under an HBW2.5/187.5 load, in Figure 9a, in the case of FR sample, some cracks can be observed in the coating, heading towards the interface, whereas in Figure 9b, for the VR sample, no cracks can be identified along the interface or in the coating, respectively. This aspect denotes that even though it presents a slightly lower hardness, the VR sample is a dense and ductile coating, not generating cracks even under a considerable load. Moreover, in the case of VR sample, the strong metallurgical bond strengthens the interface, improving the mechanical properties of the coating-substrate region.

### 3.3. Tribology

**Figure 10:**
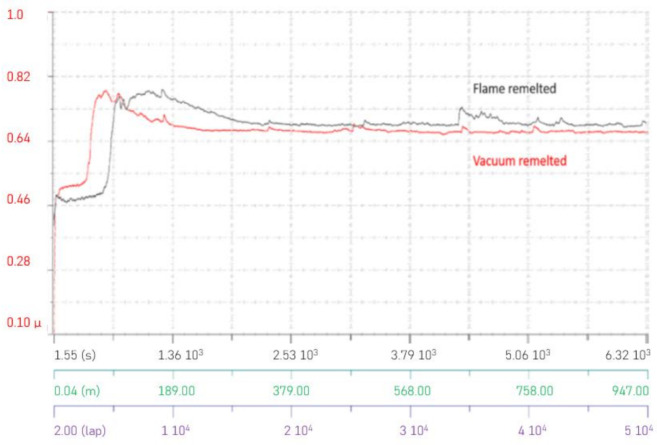
Graph highlighting the COF behavior.

The tribological behavior evaluation of the coating consisted in measuring the coefficient of friction (COF) for the remelted samples (both flame and vacuum remelted) and calculating the wear rate of the samples and their counterparts. The COF, as presented in Figure 10, reached in the steady-state phase a constant mean value of approximately 0.65, a comparable value being also reported by Wang et al. [25] for similar coatings. The only difference in the analysis of the COF is that the VR coating reached a faster steady-state slightly after around 190 m, while the FR one reached a steady-state after a distance of approximately 250 m. 

**Figure 11:**
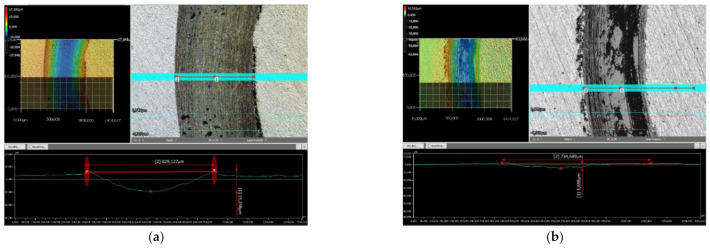
Sliding track of: FR (**a**) and VR (**b**) remelted samples.

Although the COF did not present important differences, the wear behavior of the samples was significantly different. Figure 11a,b shows the wear tracks of the samples, with a depth of 15.25 µm for the FR sample and three times smaller for the VR one. The worn cap diameter also varied, since the measured value after the dry sliding test was 809 µm for the FR sample and 689 µm for the VR one. The measured depths and diameters directly influence the wear rate coefficients, as presented in Table 5. The wear rates of the sample and counterpart are 50% lower in the case of the VR specimen. This fact is due to the hard phases containing boron, namely CrB and Ni_3_B, which are present in a larger quantity in the VR specimens, aiding against the formation of deep wear tracks and consequently increasing the wear resistance of the coating.

### 3.4. Corrosion Behavior

**Figure 12:**
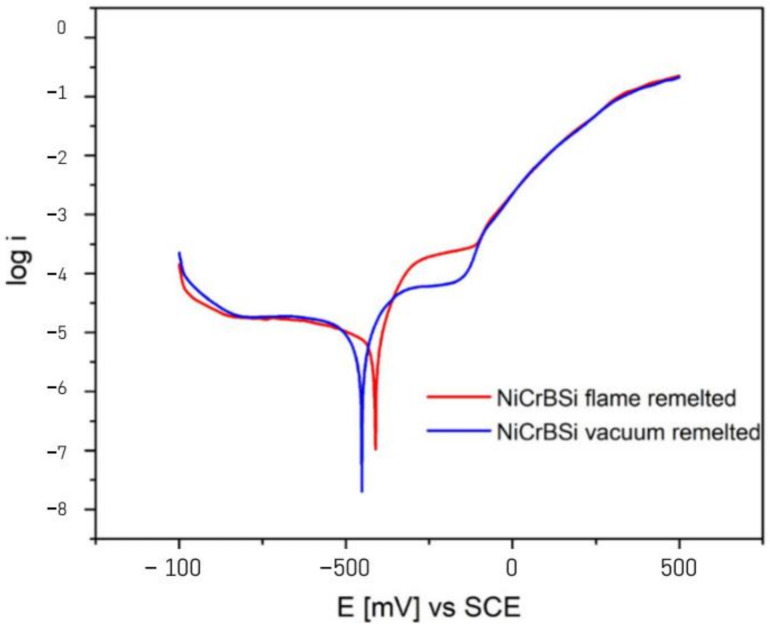
Polarization behavior of the remelted samples.

**Figure 13:**
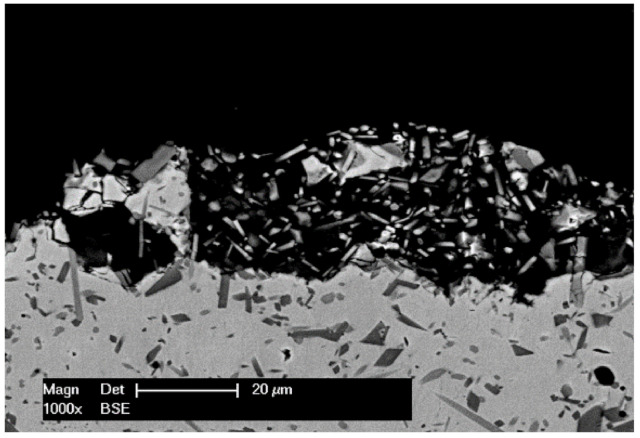
BSE micrograph of the VR corroded sample.

Bergant et al. have previously shown that the Ni-based heat-treated coatings exhibit a better corrosion behavior with a factor of 10, when compared to the as-sprayed ones [24]. Thereby, Figure 12 presents the polarization curves of the remelted specimens, while Table 6 shows the corrosion current density *i_corr_* and corrosion potential *E_corr_* estimated using the Tafel extrapolation method. Looking at the *i_corr_*, it is important to mention that the VR sample exhibited a lower value than the one obtained in the case of FR sample. Corrosion starts especially in regions with voids, and the process develops faster for the FR compared to the VR samples. Examining the polarization curves, the behavior in the anodic region is similar. The VR sample presents though a slightly longer repasivation plateau between −333 and −164 mV vs. SCE, while the FR sample indicates a similar, but shorter plateau, between −237 and −119 mV vs. SCE. A faster and longer repasivation region of the VR specimen shows improved corrosion behavior of the investigated material in the tested medium. Starting from −100 mV vs. SCE up to more positive potentials, the curves overlap on the anodic region, presenting the same corrosion behavior. Figure 13 presents a BSE micrograph of the cross-sectioned corroded VR sample, showing that the γ-Ni phase dissolved during the corrosion tests, leaving the Ni_3_B and CrB blocky phases detaching from the coating.

The corrosion potential *E_corr_* presents similar values with a minimal shift of approximately 50 mV, presumably due to the different ratios of the phases in the coatings. Minimal shifts of up to 100 mV were also observed by Makuch [26] when investigating Ni-based materials with different Cr content.

## 4. Conclusions

According to the DTA data, the fusing temperature of 1050 °C (situated approximately 20 °C under the liquidus temperature of the powder) was applied in a vacuum furnace to obtain a dense coating, with low porosity, satisfying roughness, and a strong metallurgical bond.

Although the HV0.3 microhardness of the flame remelted sample was 12% higher than that of the vacuum remelted one, the latter displayed a better adhesion to the substrate and it presented a crack-free coating when subjected to HBW indentations, an aspect which reflects a low degree of internal residual stresses, which were eliminated during the thermal post treatment.

The coefficient of friction in the case of vacuum remelted samples had stabilized earlier, offering a longer steady-state period compared to flame remelted specimens. The wear rate of the vacuum remelted sample was almost two times lower than that of the flame remelted one, a fact which can be attributed to the larger amount of hard CrB and Ni_3_B phases. The corrosion behavior of the vacuum remelted sample is slightly superior in comparison with the tested flame remelted sample, exhibiting lower current density, especially in the passivation region.

## Figures and Tables

**Table 1:** Chemical composition of the powder.

Ni (%)	Cr (%)	B (%)	Si (%)	Fe (%)	C (%)
balance	6	1	4	1.5	0.3

**Table 2:** Deposition parameters.

Parameters	Materials/Conditions/Values
**Surface roughening**	-
Surface roughening method	Grit blasting
Roughening material	Chilled iron grit
Surface roughness after roughening	min. 75 µm
**Flame spraying gun producer**	Metatherm
Flame generation	-
Fuel gas	Acetylene (C_2_H_2_)
Secondary gas	Oxygen (O_2_)
Flame stoichiometry C_2_H_2_:O_2_	1:2
Substrate temperature	≈105 °C
Spraying temperature	≈2850 °C
Fusion temperature	≈1100 °C
Particle velocity	100 m s^−1^
**Coating deposition**	-
Powder feed rate	2.5 kg h^−1^
Stand-off distance	120 mm.
Propelling gas	Purified air
Relative gun motion	Translation over the samples

**Table 3:** Pin-on-disk test parameters.

Static Counterpart	Radius (mm)	Linear Speed (cm s^−1^)	Normal Load (N)	Laps	Total Distance (m)	Test Duration (s)
WC-Co ball	3	15	10	50,000	947	6300

**Table 4:** Roughness and porosity average measurements of the as-sprayed, FR and VR samples.

Sample	*Ra* (µm)	*Rz* (µm)	Porosity (%)
As-sprayed	18.82	99.34	7.55
FR	7.32	48.81	0.6
VR	5.02	36.63	0.15

**Table 5:** Wear rate of the samples and counterparts.

Sample	Sample Wear Rate (mm^3^ N^−1^ m^−1^)	Counterpart Wear Rate (mm^3^ N^−1^ m^−1^)
FR	1.279 × 10^−5^	7.18 × 10^−7^
VR	0.705 × 10^−5^	3.91 × 10^−7^

**Table 6:** Corrosion current densities and potential of the samples.

Sample	*i_corr_* (A cm^−2^)	*E_corr_* (mV)
FR (red)	0.60 × 10^−5^	−412
VR (blue)	0.44 × 10^−5^	−453

## Data Availability

The data reported in this study are available from the authors upon request.
